# BST2 Drives Epithelial Ovarian Cancer Progression via Macrophage M2 Polarization, Neural Remodeling, and Immunosuppressive Microenvironment Formation

**DOI:** 10.1155/humu/8719836

**Published:** 2025-11-13

**Authors:** Limin Zhang, Xiaoli Huang, Shaoyu Wang, Shaozhan Chen, Jinhua Wang, Lihong Chen, Pengming Sun

**Affiliations:** ^1^College of Clinical Medicine for Obstetrics & Gynecology and Pediatrics, Fujian Medical University, Fuzhou, Fujian, China; ^2^Department of Obstetrics and Gynecology, The First Affiliated Hospital, Fujian Medical University, Fuzhou, Fujian, China; ^3^Department of Obstetrics and Gynecology, National Regional Medical Center, Binhai Campus of The First Affiliated Hospital, Fujian Medical University, Fuzhou, Fujian, China; ^4^Laboratory of Gynecologic Oncology, College of Clinical Medicine for Obstetrics & Gynecology and Pediatrics, Fujian Maternity and Child Health Hospital, Fujian medical University, Fuzhou, Fujian, China; ^5^Fujian Clinical Research Center for Gynecologic Oncology, Fujian Maternity and Child health Hospital (Fujian Obstetrics and Gynecology Hospital), Fuzhou, Fujian, China

**Keywords:** BST2, epithelial ovarian cancer, macrophages, neurons

## Abstract

**Background:**

Epithelial ovarian cancer (EOC) ranks as the most lethal of gynecological cancers. Despite advances in therapeutic interventions that have marginally extended survival rates, the early detection and management of EOC pose significant hurdles. Consequently, identifying novel therapeutic targets is imperative for enhancing the survival outcomes of patients afflicted with this malignancy.

**Purpose:**

This research is aimed at exploring the functions of Bone Marrow Stromal Antigen 2 (BST2) in the pathogenesis of EOC and their influence on macrophage polarization, evaluating their viability as targets for immunotherapy.

**Methods:**

Gene expression profiles and clinical data of EOC patients were retrieved from the TCGA repository to develop prognostic models centered on BST2. The expression patterns of BST2 in HGSOC cell lines were quantified via RT-qPCR and Western blot analyses. The impact of BST2 on the proliferative, migratory, and invasive capacities of EOC cells was assessed through gene silencing and gene overexpression experiments.

**Results:**

Elevated levels of BST2 expression were observed in EOC tissues, correlating with adverse prognostic indicators. Enhanced BST2 expression facilitated EOC cell growth, motility, and invasiveness, whereas BST2 suppression mitigated these oncogenic attributes. In vivo assessments revealed that BST2 augmentation modified the macrophage phenotypes within grafted ovarian tumors, with BST2 diminution reversing these effects.

**Conclusion:**

The findings propose that BST2 acts as a pivotal facilitator in the progression of ovarian carcinoma. The expression metrics of BST2 may serve as prognostic markers for patient outcomes in EOC. These findings suggest that BST2 is a key promoter of ovarian cancer progression, and its expression may serve as a prognostic marker. The mechanisms uncovered, including the modulation of macrophage polarization and neural marker expression, indicate that targeting BST2 represents a potential future strategy for immunotherapy in EOC.

## 1. Introduction

Ovarian cancer remains a major challenge in gynecologic oncology [1, 2]. Current treatments consist of surgery and chemotherapy. As the disease is often diagnosed at an advanced stage, these therapies are typically applied to patients with late-stage cancer [3, 4]. The late diagnosis and complicated symptoms result in a low survival rate for patients [5]. Currently, the 5-year survival rate is still below 45%, mainly due to the late diagnosis, as well as chemotherapy resistance and relapse [6]. Epithelial ovarian cancers (EOCs) account for 90% of ovarian cancers and include serous, endometrioid, clear cell, and mucinous carcinomas [7]. Among them, serous carcinoma is the most common subtype, accounting for about 70% of EOC [7]. Histologically, about 90% of ovarian cancers occurred through the transformation of epithelial cells, and high-grade serous carcinoma (HGSOC) is recognized as the most common type of EOC [8, 9]. Hence, the pursuit of novel therapeutic strategies and an enhanced comprehension of the fundamental biological processes is required to ameliorate the prognosis for individuals with EOC, the most common ovarian cancer.

Macrophages are a major component of the tumor microenvironment (TME), comprising up to 40% of infiltrating inflammatory cells [10]. These cells display significant functional plasticity, polarizing into two primary phenotypes: proinflammatory (M1) macrophages and anti-inflammatory (M2) macrophages, whose expression is orchestrated by distinct cytokines [11, 12]. The M2 phenotype, in particular, is associated with poor prognosis as it promotes tumor invasion, angiogenesis, and an immunosuppressive environment [13]. Macrophages stand out as formidable immune effector cells, with their functional versatility manifesting in both tumoricidal and tumor-promoting roles across various contexts. This versatility has catalyzed substantial endeavors to either diminish or redirect the orientation of tumor-associated macrophages [14]. A study by Sheng et al. found that high expression of the membrane-bound protein Copine 1 (CPNE1) in EOC correlated with poor prognosis and, by promoting the M2 type of macrophage polarization, provided a potential therapeutic target for EOC [15]. Regulatory T cells (Tregs), recognized as the predominant immune suppressive cell subset, play a pivotal role in tumor immune evasion through their infiltration and functional maintenance within tumor tissues [16]. However, the specific differences in immune cell infiltration profiles between various tumor subtypes, such as EOC and normal ovarian tissue (OV), remain poorly understood. Furthermore, the potential direct molecular interactions and mutual enhancement between tumor cells and Tregs have not been fully elucidated. This part of the study is aimed at first characterizing and comparing the immune infiltration features in EOC and OV subtypes and, subsequently, investigating the key molecular interactions that may occur between tumor cells and Tregs in a Treg-dominated microenvironment [17]. A crucial aspect of this interaction involves how tumor cells, through the expression of specific surface molecules, modulate Treg infiltration and function. Therefore, identifying tumor-intrinsic factors, such as BST2, that may orchestrate this immunosuppressive axis is a key objective of this investigation.

However, different TMEs in different types of tumors differentially affect macrophage function, which is one of the reasons for the variability of targeted macrophage therapy. Therefore, further studies targeting different macrophage mechanisms in specific tumors are necessary for the development of new effective immunotherapies.

Furthermore, emerging evidence highlights the critical role of the tumor neuro-microenvironment. The intricate interplay between cancer cells and nerve fibers, often termed tumor-induced neurogenesis, can actively promote cancer cell proliferation, invasion, and metastasis. However, the specific molecular players that mediate this neuroimmune crosstalk within the EOC microenvironment remain largely unexplored, creating a knowledge gap that this study is aimed at addressing. Bone Marrow Stromal Cell Antigen 2 (BST2), a transmembrane glycoprotein initially identified as HM1.24 and later referred to as BST2 or CD317, has gained attention due to its elevated levels in multiple myeloma reported in 1994 [18]. BST2 is known for its antiviral efficacy, which is also referred to as tetherin, and its ability to impede the HIV-1 viral efflux, while the Viral Protein U counteracts this process [19]. This unique tethering mechanism, which involves modulating cell membrane organization and vesicle release, suggests that BST2's functions could be co-opted in pathological contexts beyond viral infection, such as regulating cell–cell communication and invasion in cancer. Studies indicate that BST2 expression is elevated in an array of neoplastic diseases, encompassing multiple myeloma, endometrial, and gastric malignancies, in addition to glioblastoma and primary pulmonary neoplasms [20–22]. Liu and colleagues revealed that in a human cancer cell line (SiHa) derived from cervical squamous cell carcinoma, BST2 inhibition reduced SiHa cell viability, migration, and invasive capacity. In addition, inhibition of BST2 induced an increase in M1 macrophage polarization (CD80 and CD86), while inhibiting M2 macrophage polarization (CD206 and CD163) [21]. Additionally, BST2 stimulated the progression of colorectal cancer by inducing M2 macrophage polarization [23]. In a most recent paper, CpG-binding protein CXXC Zinc Finger Protein 1 (CFP1) stimulated cell proliferation in an ovarian cancer cell line (A2780) by enhancing BST2 transcription, and BST2 inhibition could reduce cell proliferation [22]. However, the exact role of these proteins in ovarian cancer progression and associated macrophage polarization, as well as in cancer cellular apoptosis, migration, and invasion, remains to be determined.

Furthermore, emerging evidence highlights the critical role of the neuro-microenvironment in tumorigenesis. The interaction between cancer cells and nerve fibers, often termed tumor-induced neurogenesis, can actively promote cancer cell proliferation, invasion, and metastasis. However, the specific molecular players that mediate this neuroimmune crosstalk within the EOC microenvironment remain largely unexplored.

## 2. Materials and Methods

### 2.1. Immune Infiltration and Correlation Analysis

To characterize the tumor immune microenvironment, we quantified the relative fractions of 22 distinct immune cell subsets from RNA-sequencing profiles. Transcriptomic data, formatted as transcripts per million (TPM), were procured from The Cancer Genome Atlas (TCGA) for EOC samples and the Genotype–Tissue Expression (GTEx) project for OVs. The deconvolution was executed using the CIBERSORT R script (Version 1.04) with the LM22 signature gene matrix and 1000 permutations to ensure analytical robustness.

Differentially infiltrated immune cell populations between the EOC and OV cohorts were identified using the Wilcoxon rank-sum test, with results depicted as boxplots. The overall immune composition and hierarchical clustering patterns were visualized using stacked bar charts (ggplot2) and heat maps (pheatmap), respectively. Furthermore, to investigate the relationship between BST2 expression and immune cell abundance, we calculated Pearson's correlation coefficients. These associations were subsequently rendered in a correlation heat map (corrplot), wherein the strength and direction of the correlations were represented by the size and color intensity of each point (blue for positive and red for negative).

### 2.2. Workflow and Output

The primary output of the deconvolution was a data matrix quantifying the proportions of 22 immune cell types for each sample. This matrix was first analyzed to ascertain significant differences in cell populations between the EOC and OV cohorts through a Wilcoxon rank-sum test. The results were depicted graphically using boxplots, with significance levels annotated (*p* < 0.05, ⁣^∗^*p* < 0.01, and ⁣^∗∗^*p* < 0.001). Further visualizations were created to provide a comprehensive overview of the immune environment; stacked bar charts (ggplot2) were employed to show the cellular composition of individual samples, while a heat map (pheatmap) was used to display the hierarchical clustering of immune fractions, effectively discriminating the distinct infiltration signatures of the two groups.

### 2.3. Correlation Analysis Between BST2 Expression and Immune Cell Fractions

Pearson's correlation analysis was performed to systematically quantify the association between BST2 transcript levels and the proportions of infiltrating immune cell populations. The resulting correlation matrix was rendered as a heat map via the corrplot package. In this visualization, the size and color intensity of each circle encode the strength of the association, while the hue denotes its direction (blue for positive and red for negative correlations).

### 2.4. Data Collection and Preprocessing

To identify prognostic gene signatures in EOC, a multistage bioinformatic analysis was performed on transcriptomic and clinical data procured from TCGA. The initial stage involved a two-pronged screening process: identifying differentially expressed genes (DEGs) between tumor and control tissues (limma; |log2FC| > 1, *p* < 0.05) and separately identifying genes significantly correlated with both overall survival (OS) and progression-free survival (PFS) (survival; *p* < 0.05).

To discern functional relationships among these candidates, a protein–protein interaction network was constructed using the STRING database (confidence score > 0.4). The convergence of these analytical streams highlighted BST2 as a high-priority EOC risk gene. Consequently, a prognostic risk score model was developed using the glmnet package, integrating BST2 expression with clinical parameters. The predictive accuracy and robustness of this final model were then rigorously evaluated.

### 2.5. Patients and Samples

This study was conducted in full accordance with the principles of the Declaration of Helsinki and its subsequent amendments. All protocols received formal approval from the Medical Ethics Committee of The First Affiliated Hospital of Fujian Medical University (approval nos. [2015]084-1 and [2015]084-2). EOC tissues were procured from the Department of Obstetrics and Gynecology at the same institution, and written informed consent was secured from every participant prior to their inclusion in the research.

The analysis was based on a cohort of 31 paraffin-embedded EOC tissue specimens, which were surgically excised between August 2020 and March 2023 at The First Affiliated Hospital of Fujian Medical University and subsequently subjected to immunohistochemistry (IHC). The clinicopathological characteristics of the patient cohort are detailed in Table 1. A majority of the patients presented with International Federation of Gynecology and Obstetrics (FIGO) Stage I–II disease (18/31, 58.06%). The mean preoperative and postoperative Cancer Antigen 125 (CA-125) levels were 841.65 and 295.99 U/mL, respectively. Notably, while BST2 expression showed a pronounced trend toward an association with advanced FIGO stage, this correlation did not reach statistical significance (*p* = 0.075). Furthermore, no significant associations were identified between BST2 expression and any other clinicopathological parameters evaluated.

### 2.6. Cell Culture and Authentication

The human EOC cell lines SKOV3 (RRID:CVCL_0532) and OVCAR3 (RRID:CVCL_0465), alongside the nonmalignant human ovarian epithelial cell line IOSE-80, were utilized in this study. These cell lines, all derived from female ovarian epithelial tissue, were procured from Procell Life Science & Technology Co., Ltd. (Wuhan, China) between 2021 and 2022. They were maintained in either DMEM or RPMI-1640 medium (Thermo Fisher Scientific, United States) supplemented with 10% fetal bovine serum (FBS) (Gibco, United States) and incubated at 37°C in a humidified atmosphere containing 5% CO₂ [24]. The human monocytic cell line THP-1 (RRID:CVCL_0006), originating from male peripheral blood, was also sourced from Procell and cultured in RPMI-1640 containing 10% FBS and 0.05 mM 2-mercaptoethanol (Sigma-Aldrich, Merck KGaA). For differentiation into M0 macrophages, THP-1 cells were stimulated for 24 h with 100 ng/mL phorbol 12-myristate 13-acetate (PMA).

Rigorous quality control measures were implemented to ensure the validity of all cell lines. Routine screening using the MycoAlert Mycoplasma Detection Kit (Lonza, Switzerland) consistently confirmed that all cultures were free of mycoplasma contamination. Furthermore, the authenticity of the SKOV3 and OVCAR3 lines was verified by short tandem repeat (STR) profiling. The analysis, performed with GeneMapper ID-X Version 1.5 software (Thermo Fisher Scientific), demonstrated 100% concordance with reference profiles from the ATCC and DSMZ cell repositories. All cell lines were cross-referenced against the ICLAC database (Version 2024), which verified that none are listed as misidentified or cross-contaminated.

### 2.7. Lentiviral Construction and Transfection

The specific nucleotide sequences used for BST2 overexpression and for the shRNA-mediated knockdown of BST2 are detailed in Figure S1.

To engineer BST2 overexpression (OE), lentiviral particles (LV-0683) were generated using the pLV-EF1a-BST2-CMV-EGFP-T2A-Puro vector. These particles were subsequently transduced into OVCAR cells following standard protocols to establish stable overexpression. For gene silencing, a different lentiviral construct (LV-0684), based on the pLVX-U6-shRNA(BST2)-CMV-Puro vector, was employed. This construct expresses an shRNA designed to specifically target the 3⁣′ untranslated region (UTR) of BST2 messenger RNA (mRNA) and was used to create stable knockdown cell lines in SKOV3 cells.

### 2.8. Quantitative Real-Time Reverse Transcription-Polymerase Chain Reaction (qRT-PCR) Analysis

The relative mRNA expression of target genes was quantified using qRT-PCR. Initially, total RNA was isolated from cell samples with TRIzol reagent (Vazyme, China). Subsequently, complementary DNA (cDNA) was synthesized from the extracted RNA templates using the PrimeScript RT kit (Vazyme, China).

The qRT-PCR amplification was conducted using Green Master Mix (TaKaRa, China) according to the manufacturer's instructions. All oligonucleotide primers were synthesized by DynaPro Bio, utilizing the following sequences: Foxp3: forward 5⁣′-TGTGCTCAGAGGACACCTCTG-3⁣′, reverse 5⁣′-TGGGAAGAGACAGGAGTAGAG-3⁣′; IL-10: forward 5⁣′-AACTGCACCCACTTCTGCC-3⁣′, reverse 5⁣′-TTCAGTCCAGTGGCAAAGG-3⁣′; and BST2: forward 5⁣′-GTCACCCATCTCCTGCAACA-3⁣′, reverse 5⁣′-TCTCTCCCTCAAGCTCCTCC-3⁣′. For data analysis, the expression of each target gene was normalized to the endogenous reference gene, GAPDH. The relative fold-change in gene expression was calculated based on the 2^−ΔΔCt^ method [25]. The results presented are the mean values derived from a minimum of three independent experiments.

### 2.9. CCK-8 Assay for Cell Viability

For this assay, 5 × 10^3^ cells were seeded into each well of a 96-well plate. The CCK-8 solution was then added at specified time points—0, 24, 48, 72, and 96 h. Following incubation, the optical density was measured at a wavelength of 450 nm. To ensure the reliability of the data, the entire experiment was performed in triplicate [26]. This procedure was replicated thrice to ensure reliability.

### 2.10. Colony Formation Assay

To induce spheroid formation, cells were cultured in ultralow attachment 24-well plates. After a 6-day incubation period, the resulting spheroids were imaged using an EVOS FL Automated Imaging System (Life Technologies). For each biological replicate, images were captured from at least three different wells to ensure representative data [27]. This procedure was replicated thrice to ensure reliability.

### 2.11. Flow Cytometry for Macrophage Subset Analysis

Flow cytometric analysis was performed using a FACScan instrument to quantify the polarization of macrophages into M1 and M2 phenotypes. The cells for this analysis were sourced from both a coculture system and dissociated tumor tissues. Discrimination between these macrophage subpopulations relied on immunophenotyping for the canonical surface markers CD86 and CD206, which, respectively, identify the M1 and M2 phenotypes.

The gating strategy first isolated single, viable cells by excluding debris and doublets using forward scatter/side scatter (FSC/SSC) parameters, followed by the removal of dead cells via a viability dye. Within this refined cell population, M1 macrophages were defined as the CD86^+^ CD206^−^ fraction, whereas M2 macrophages were identified as the CD86^−^ CD206^+^ fraction. Notably, any cells coexpressing both markers (CD86^+^ CD206^+^) were excluded from this strict M1/M2 classification, as they may represent a transitional or mixed-phenotype state [28].

### 2.12. Detection of Apoptosis and Cycle by Flow Cytometry

Cells were first cultured to approximately 80% confluency, at which point they were harvested and dissociated into a single-cell suspension via trypsinization. For the assessment of apoptosis, the cell suspension was stained with an Annexin V-FITC and propidium iodide (PI) solution (Procell, China) in the dark. For cell cycle analysis, a separate aliquot of cells was incubated with 1 *μ*L of RedNucleus I solution (Procell, China) for 10 min at 37°C. Both analyses were subsequently performed on a BD flow cytometer [29].

### 2.13. Transwell Assay

In this experiment, 6 × 10^4^ cells were suspended in serum-free medium and seeded into the upper chamber of an 8 *μ*m pore size insert (Corning). The lower chamber was filled with RPMI-1640 medium containing 10% FBS, which functioned as a chemoattractant. Following a 48-h incubation period, nonmigratory cells were carefully removed from the upper surface of the membrane. Cells that had successfully migrated through the pores to the lower surface of the membrane were then fixed with formaldehyde and stained with crystal violet (Solarbio, China). Finally, the extent of cell migration was determined through microscopic imaging and subsequent quantification [30].

### 2.14. Western Blot Analysis

For Western blot analysis, total cellular proteins were first extracted using RIPA buffer (Thermo Fisher Scientific, United States) fortified with protease inhibitors (KeyGene Biotech, China). The protein concentration of the resulting lysates was determined by the bicinchoninic acid (BCA) method.

Equal quantities of protein for each sample were then resolved by electrophoresis on a 10% SDS-polyacrylamide gel and subsequently transferred to a polyvinylidene difluoride (PVDF) membrane (Thermo Fisher Scientific). To prevent nonspecific antibody binding, the membranes were blocked for 60 min in a Tris-buffered saline solution containing 5% nonfat milk.

For immunodetection, the membranes were incubated overnight at 4°C with primary antibodies directed against BST2 (1:1000 dilution; Abcam, United States) and *β*-actin (1:1000 dilution; Abcam, United States), with the latter serving as a loading control. Following this, the membranes were washed and probed for 1 h at room temperature with an appropriate HRP-conjugated secondary antibody. After three final washes, the immunoreactive protein bands were visualized using an enhanced chemiluminescence (ECL) substrate and documented with an Odyssey Imaging System. The relative protein expression was quantified by measuring the optical density of the bands with ImageJ software, normalizing the signal from the target protein to that of the corresponding *β*-actin control.

### 2.15. Enzyme-Linked Immunosorbent Assay (ELISA)

The concentrations of the cytokines IL-10, IL-4, and CCL22 within the cell culture supernatants were quantified using commercial ELISA kits (Beyotime, China). The procedure was executed in strict accordance with the manufacturer's protocol. The optical density of each sample was measured at 450 nm using a microplate reader, and the final concentration for each cytokine was determined by interpolation from a standard curve generated during the same assay.

### 2.16. Tumor Xenografts

All animal procedures were performed under the ethical approval of the Ethics Committee for Laboratory Animals of Fujian Medical University (IACUC FJMU 2024-0044) and were conducted in strict compliance with the ARRIVE guidelines.

For the in vivo xenograft model, female BALB/c nude mice (4–6 weeks old, ~20 g; Charles River) were first allowed a 1-week acclimatization period at 25°C. To initiate tumor growth, mice were anesthetized via intraperitoneal injection of pentobarbital sodium (30–90 mg/kg; Bio-Techne, United States). Subsequently, a suspension of 5 × 10^6^ ovarian cancer cells in 0.1 mL of PBS was subcutaneously inoculated into the right anterior axilla of each animal.

The mice were then randomly allocated into four distinct experimental cohorts (*n* = 6 per group) to investigate the function of BST2. Two cohorts were inoculated with either control SKOV3 cells or SKOV3 cells engineered for stable BST2 knockdown (SKOV3 + BST2 KD). The other two cohorts received either control OVCAR3 cells or OVCAR3 cells with stable BST2 overexpression (OVCAR3 + BST2 OE).

Throughout the experiment, the body weight and tumor dimensions (length, *x*, and width, *y*) of each mouse were recorded every 2 days. Tumor volume (*V*) was calculated using the standard formula: *V* = 0.5 × *x* × *y* [2]. At the study's conclusion, all mice were euthanized by cervical dislocation as stipulated by the approved ethical protocols.

### 2.17. Hematoxylin and Eosin (H&E) Staining

For histological examination, tumor tissues were excised from the mice immediately posteuthanasia and fixed in 4% paraformaldehyde for 24 h. Following fixation, the specimens were processed through a standard dehydration protocol and subsequently embedded in paraffin blocks. These blocks were then sectioned into 5-*μ*m-thick slices. The resulting tissue sections were stained with H&E to visualize cellular and tissue morphology. Finally, the stained slides were examined, and representative images were captured using an inverted microscope.

### 2.18. IHC Detection

For IHC analysis, the prepared paraffin sections first underwent deparaffinization in xylene, followed by rehydration through a graded ethanol series. To block endogenous enzyme activity, the sections were treated with 3% hydrogen peroxide for 15 min at room temperature. Heat-induced epitope retrieval was then performed by immersing the slides in a 0.01 M citrate buffer (pH 6.0) and heating them in a microwave for 10 min. Subsequently, the tissue sections were incubated overnight at 4°C with specific primary antibodies targeting BST2 (1:2000; Cell Signaling Technology), CD163 (1:500; Cell Signaling Technology), CD86 (1:300; Bioss), or Ki67 (1:200; Thermo Fisher Scientific). Following incubation with the appropriate secondary antibodies, the signal was visualized using a DAB chromogen kit (GTVision Detection System/Mo&Rb).

The staining results were quantified by counting the number of DAB-positive cells per slide. To further assess the overall protein expression while accounting for staining heterogeneity, an *H*-score was calculated. This score was determined using the formula: *H* − score = (3 × %of strongly stained cells) + (2 × %of moderately stained cells) + (1 × %of weakly stained cells). This calculation yields a final score ranging from 0 to 300, providing a comprehensive semiquantitative value for the staining intensity across the tissue.

### 2.19. Tissue Immunofluorescence

For immunofluorescence staining, tumor tissues harvested from mice bearing EOC xenografts were fixed in 4% paraformaldehyde and processed into paraffin-embedded sections. Following deparaffinization, antigen retrieval was conducted by heating the tissue sections in an EDTA buffer (pH 8.0) within a microwave oven.

To minimize nonspecific signals, the sections were blocked with a 4% BSA solution for 30 min. The tissues were then incubated overnight at 4°C with primary antibodies targeting neuron-specific enolase (NSE) and glial fibrillary acidic protein (GFAP) (1:200 dilution; Servicebio, China). After washing, the sections were probed for 50 min in a light-protected environment with a FITC-conjugated sheep antirabbit IgG secondary antibody (1:2000 dilution). Cell nuclei were subsequently counterstained with 4⁣′,6-diamidino-2-phenylindole (DAPI). Finally, the fluorescent intensity of NSE and GFAP signals was visualized and quantified using an Olympus fluorescence microscope.

### 2.20. Statistical Analysis

Differences between the two independent groups were evaluated using a two-tailed unpaired Student's *t*-test. For the analysis of categorical data, such as comparing the proportions of low versus high BST2 expression, the chi-square (*χ*^2^) test or Fisher's exact test was applied as appropriate. The relationship between BST2 expression levels and other variables was assessed using Spearman's rank correlation test.

All statistical computations were performed using SPSS (Version 22.0) and GraphPad Prism (Version 9.0) software.

## 3. Results

### 3.1. Elevated Levels of BST2 in EOC Correlate With Adverse Patient Outcomes

**Figure 1:**
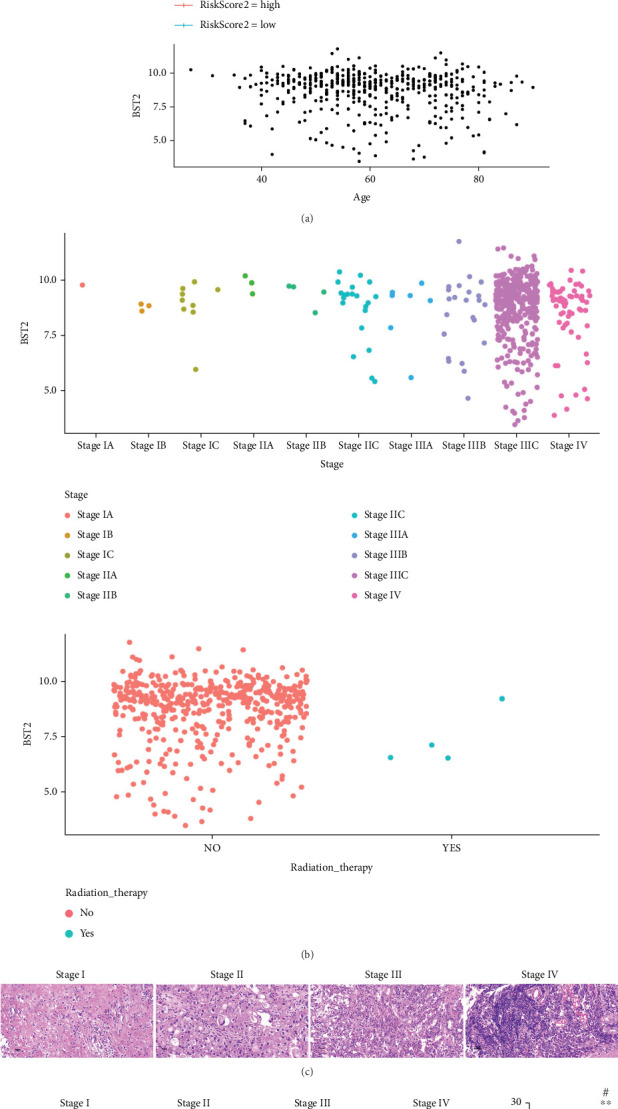
Clinical implications of BST2 overexpression in EOC. (a) Stratification of patients into high- and low-risk groups based on factor risk score regression for survival analysis. (b) Associative analysis linking BST2 expressions with clinical staging, patient age, and radiotherapy status. (c) Histopathological evaluation by H&E staining to observe the histopathological changes of EOC tissues, scale bars = 50* μ*m. (d) IHC to detect the expression of BST2 in EOC tissues. Top row: images at 5× magnification, scale bar = 200* μ*m; bottom row: images at 20× magnification, scale bar = 50* μ*m. (⁣^∗^*p* < 0.05 and ⁣^∗∗^*p* < 0.01 compared with the Stage I group; #*p* < 0.05 compared with the Stage II group).

Research has consistently indicated that the gene BST2 is overexpressed in diverse cancerous tissues, contributing to the advancement of the disease [20, 31]. To elucidate the clinical and pathological significance of BST2 in EOC, an analysis of RNA-sequencing and clinical data from TCGA was conducted. This investigation established that BST2 transcription is significantly upregulated in EOC tissues. Crucially, this elevation in BST2 expression was a powerful predictor of poor prognosis; survival analysis demonstrated that patients within the high-expression cohort had markedly diminished survival rates (Figure 1a, *p* < 0.0001). BST2 expression was subsequently correlated with key clinical parameters. A strong positive association was found between BST2 levels and advancing cancer stage. While no significant overarching correlation with age was detected, expression was most pronounced in the 50–70 age demographic. Notably, higher BST2 expression was observed in patient records lacking chemotherapy administration; however, it should be noted that this correlation may be influenced by confounding variables such as disease severity at presentation (Figure 1b). To validate these transcriptomic findings at the protein level, histological analyses of EOC patient tissues were performed. H&E staining revealed a concomitant increase in nuclear heterogeneity, mitotic activity, and cellular disorganization with advancing tumor stage (Figure 1c). Correspondingly, IHC analysis confirmed a progressive rise in BST2 protein expression. The median BST2 *H*-scores for Stages I, II, III, and IV were 9, 11, 17, and 27, respectively, with Stage III and IV tumors showing a significantly higher rate of positivity compared to their Stage I and II counterparts (Figure 1d). Collectively, these findings establish a robust link between elevated BST2 expression in EOC and adverse clinical outcomes, positioning BST2 as a promising biomarker for prognostication and a potential therapeutic target.

### 3.2. BST2 Expression Is Upregulated in EOC Cell Lines SKOV3 and OVCAR3

**Figure 2:**
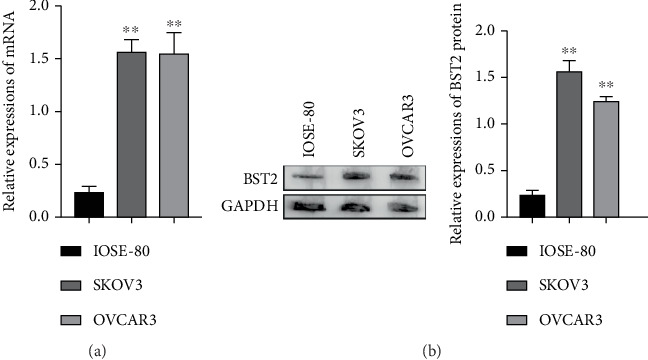
BST2 expression is upregulated in epithelial ovarian cancer cell lines SKOV3 and OVCAR3. (a) qRT-PCR for BST2 mRNA; (b) Western blot for BST2 protein expression level (⁣^∗∗^*p* < 0.01).

To corroborate the findings from patient tissues at a cellular level, the expression of BST2 was quantitatively assessed in vitro. A comparison between the nonmalignant human ovarian epithelial cell line IOSE-80 and two EOC cell lines, SKOV3 and OVCAR3, was performed. Both qRT-PCR and Western blot analyses consistently demonstrated a substantial upregulation of BST2 at the mRNA and protein levels in the cancerous SKOV3 and OVCAR3 cells relative to the IOSE-80 control (Figure 2a,b). This pronounced overexpression in EOC cell lines further implicates BST2 in the mechanisms of ovarian carcinogenesis, reinforcing its potential as a viable biomarker and therapeutic target.

### 3.3. BST2 Upregulation Promotes Malignant Behavior in EOC Cell Lines

**Figure 3:**
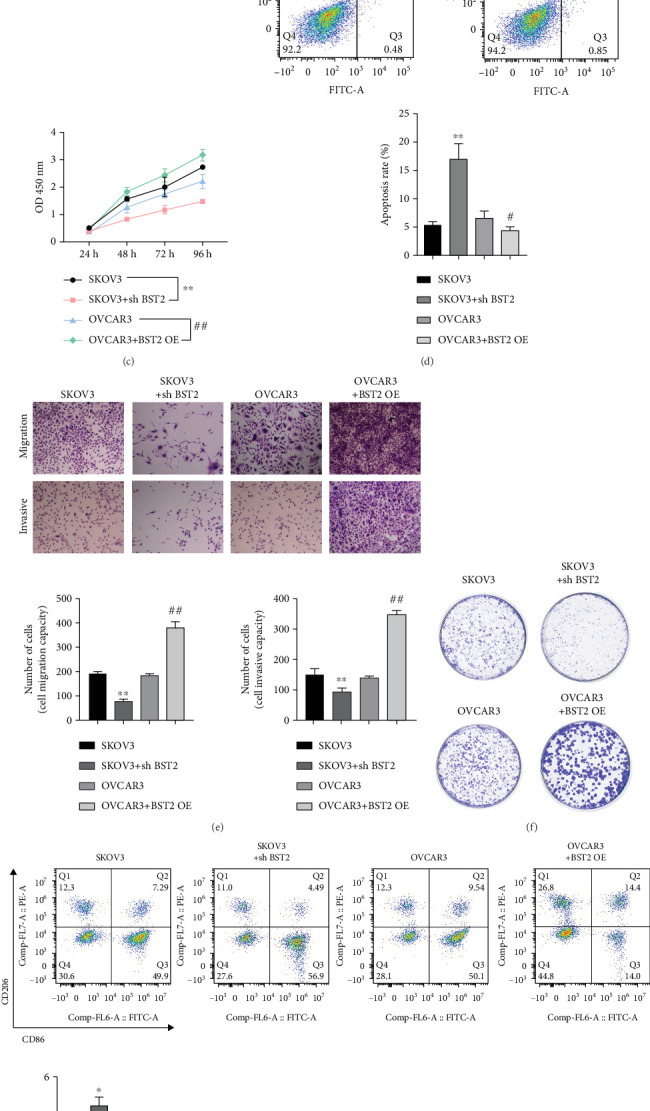
BST2 regulation and its impact on epithelial ovarian cancer cell dynamics. (a) qRT-PCR analysis confirming the successful knockdown of BST2. (b) Western blot assessment to evaluate the knockdown efficiency of BST2. (c) CCK-8 assays conducted to measure cell proliferation post-BST2 knockdown in SKOV3 cells and following BST2 overexpression in OVCAR3 cells. (d) Flow cytometry employed to ascertain the influence of BST2 expression on apoptosis rates within SKOV3 and OVCAR3 cell lines. (e) Transwell assays executed to gauge the impact of BST2 expression on the migratory and invasive capacities of SKOV3 and OVCAR3 cells. (f) Colony formation. (g) Flow cytometry to detect the M1/M2 ratio. (h, i) ELISA to detect IL-10 and IL-4 content in cell supernatants. (⁣^∗^*p* < 0.05, ⁣^∗∗^*p* < 0.01, and ⁣^∗∗∗^*p* < 0.001 compared with the SKOV3 group; #*p* < 0.05 and ##*p* < 0.01 compared with the OVCAR3 group.)

To define the functional role of BST2 in EOC, its expression was experimentally modulated in two distinct cell lines. Stable BST2 knockdown was achieved in SKOV3 cells, which have high endogenous expression, while BST2 was overexpressed in OVCAR3 cells, which have low endogenous levels. These modifications were confirmed at both the transcript and protein levels via qRT-PCR and Western blot (Figure 3). The subsequent functional assays demonstrated that BST2 is critical for maintaining malignant phenotypes. BST2 knockdown significantly suppressed cell proliferation, as measured by the CCK-8 assay, and reduced the capacity for colony formation. Conversely, BST2 overexpression augmented both of these oncogenic properties (Figure 3). Furthermore, Transwell and scratch wound assays revealed that BST2 depletion markedly impaired the migratory and invasive capabilities of EOC cells (Figure 3). Consistent with a prosurvival function, flow cytometry analysis showed that silencing BST2 led to a pronounced increase in the rate of apoptosis (Figure 3). Next, the influence of BST2 on the TME was investigated using an in vitro coculture system with M0 macrophages. Flow cytometry revealed that EOC cells with BST2 knockdown induced a significantly lower M1/M2 macrophage ratio, whereas BST2-overexpressing cells prompted a higher ratio (Figure 3). Analysis of secreted factors by ELISA showed that the sh-BST2 group produced lower levels of the M2-polarizing cytokines IL-4 and IL-10, while the BST2-OE group secreted higher amounts (Figure 3). Taken together, these results indicate that BST2 functions as an oncogenic driver in EOC by promoting cell proliferation, motility, and survival while also modulating macrophage polarization.

### 3.4. BST2 Overexpression Promotes Macrophage M2 Polarization and Transplant Tumor Growth in Ovarian Cancer Tissues

**Figure 4:**
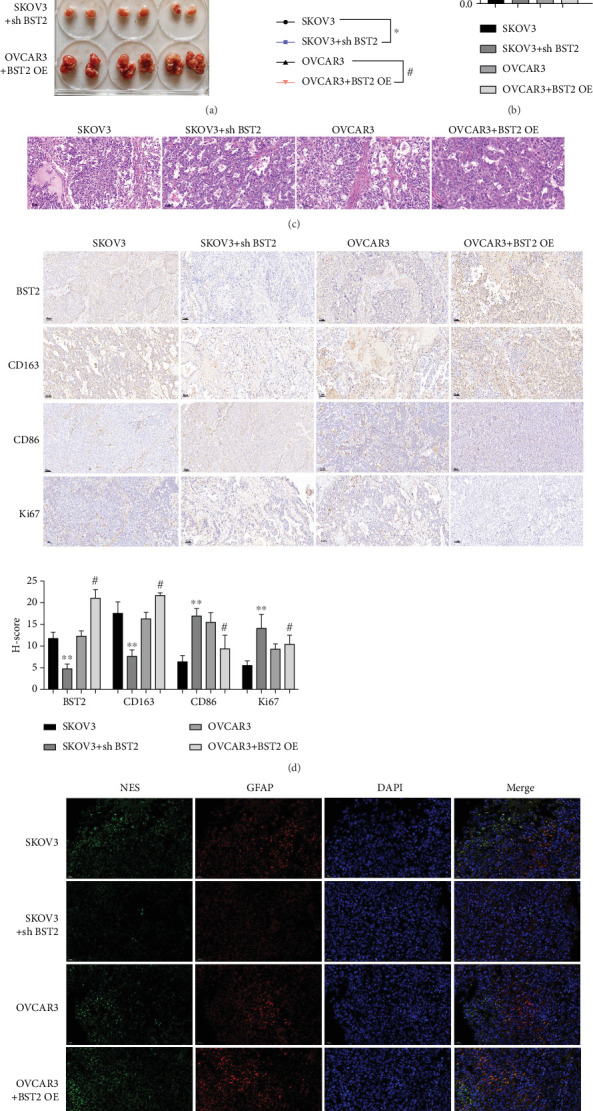
Effect of BST2 expression on the growth of EOC transplanted tumors. (a) Calculation of tumor volume (in a 6-well plate, inner diameter at the bottom of the hole is about 35 mm). (b) Tumor weight recording. (c) Histological staining to observe the histological changes of transplanted tumor tissues in mice. (d) IHC staining to detect the expression of BST2, macrophage surface marker protein, and Ki67, scale bars = 50* μ*m. (e) Multiplex immunofluorescence assay to determine the expression of neuromarkers NSE (green) and GFAP (red) and quantification of mean fluorescence intensity in transplanted tumors, scale bars = 20* μ*m. (⁣^∗∗^*p* < 0.01 compared with the SKOV3 group; #*p* < 0.05 and ##*p* < 0.01 compared with the OVCAR3 group.)

To validate the protumorigenic role of BST2 in vivo, a xenograft model was established by implanting immunodeficient mice with EOC cells featuring either BST2 knockdown or overexpression. The results demonstrated that tumors derived from BST2-overexpressing cells grew to a significantly greater volume and mass compared to those in the control and knockdown cohorts (Figure 4a,b). Histological examination of these larger tumors confirmed distinct pathological features and revealed markedly higher expression of the proliferation marker Ki67, indicating enhanced cell division (Figure 4c). Analysis of the TME showed that BST2 actively reshapes the immune landscape. IHC staining of xenograft tissues found that BST2 overexpression was associated with an increased density of CD163-positive M2 macrophages and a concurrent reduction in CD86-positive M1 macrophages, signifying a shift toward an immunosuppressive, protumorigenic M2 polarization (Figure 4d). Furthermore, immunofluorescence analysis uncovered a novel link to neural markers; BST2 overexpression stimulated the expression of Nestin (NES) and GFAP, an effect that was abrogated by BST2 knockdown (Figure 4e). In summary, the in vivo data establish that BST2 not only drives the physical growth of EOC tumors but also remodels the TME and is associated with the induction of specific neural markers, solidifying its role as a key factor in EOC progression.

### 3.5. BST2 Promotes M2 Polarization of Macrophages and Involves the Activation of the IL-4/STAT6 Pathway

**Figure 5:**
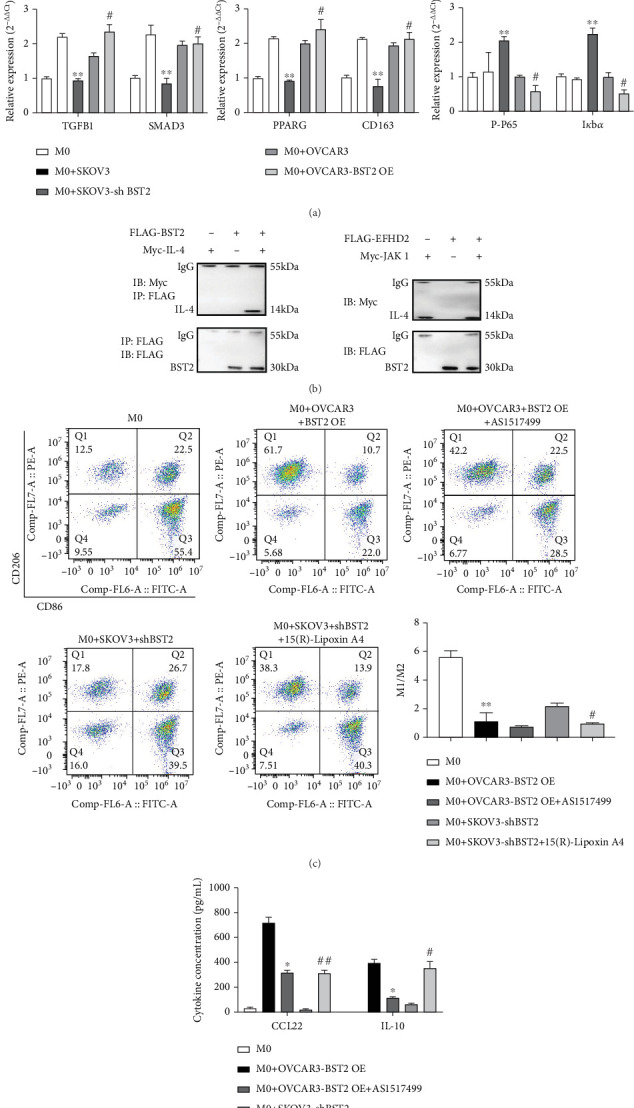
The Role of BST2 in modulating macrophage polarization and cytokine secretion via the IL-4/STAT6 pathway. (a) qRT-PCR analysis of the expression levels of immune-related genes (IL-4, STAT6, IL-10, SOCS3, TGFB1, SMAD3, PPARG, CD163, P-P65, and I*κ*B*α*) in different experimental groups (⁣^∗^*p* < 0.05 and ⁣^∗∗^*p* < 0.01 compared with the M0 + SKOV3 group; #*p* < 0.05 compared with the M0 + OVCAR3 group). (b) Immunoprecipitation analysis of the interaction between BST2 and IL-4 or JAK1. (c) Flow cytometry analysis of CD206 and CD86 expression on macrophages, indicating polarization state (M2/M1 ratio). (d) ELISA detection of CCL22 and IL-10 cytokine secretion levels in macrophage culture supernatants. (⁣^∗^*p* < 0.05 and ⁣^∗∗^*p* < 0.01 compared with the M0 + OVCAR3-BST2 OE group; #*p* < 0.05 and ##*p* < 0.01 compared with the M0 + SKOV3-sh BST2 group.)

To elucidate the molecular mechanism by which BST2 directs macrophage polarization, the expression of critical immunoregulatory pathways was quantified. qRT-PCR analysis revealed that coculture with BST2-overexpressing EOC cells prompted a significant upregulation of genes associated with M2 polarization and inflammation in M0 macrophages, including the IL-4/STAT6, IL-10, TGF-*β*, PPAR*γ*, and NF-*κ*B pathways. Conversely, coculture with BST2-knockdown cells resulted in the suppression of these same genetic programs (Figure 5a). Given the central role of IL-4/STAT6 signaling in M2 polarization, a direct interaction was investigated. Coimmunoprecipitation experiments confirmed that BST2 physically associates with IL-4, suggesting a direct regulatory role (Figure 5b). To functionally validate this axis, pharmacological interventions were used. In the BST2 overexpression setting, application of the specific STAT6 inhibitor AS1517499 effectively abrogated the M2 phenotype, leading to a significant decrease in the M2/M1 macrophage ratio and reduced secretion of the M2-associated cytokines CCL22 and IL-10. In a complementary experiment, the M2-suppressed phenotype in the BST2-knockdown group was successfully rescued by treatment with the M2 polarization inducer Lipoxin A4, which restored the M2/M1 ratio and cytokine production (Figure 5c,d). Collectively, these findings demonstrate that BST2 promotes M2 macrophage polarization by directly engaging the IL-4 signaling pathway to activate STAT6.

The expression of BST2 is closely related to the formation of an antitumor immune microenvironment in ovarian cancer.

**Figure 6:**
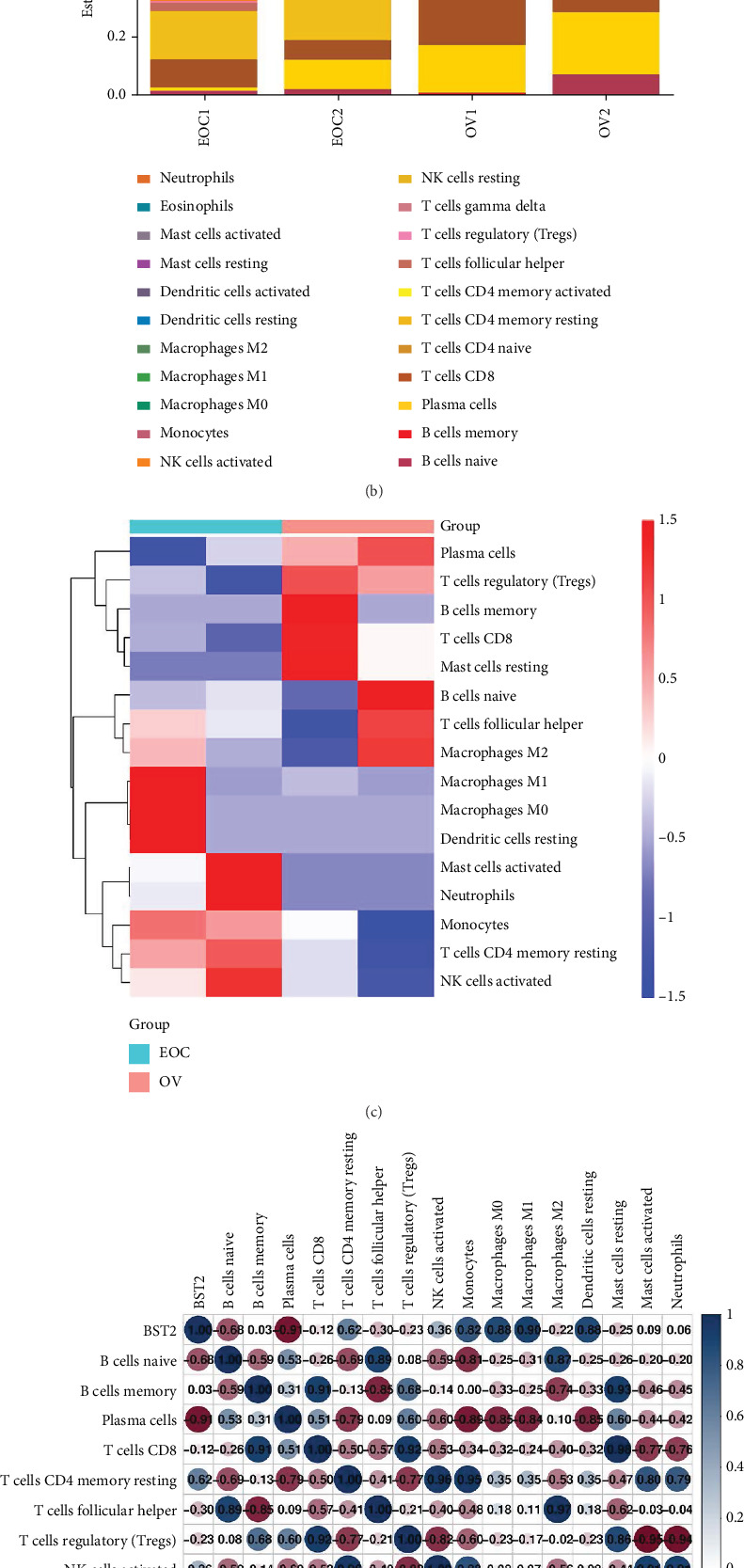
Impact of BST2 expression on immune cell infiltration and Treg function in ovarian cancer. (a) Single-cell analysis comparing the immune cell composition in the OV and EOC groups, showing increased proinflammatory cells (M1 macrophages, activated mast cells, and CD8+ T cells) and decreased immunosuppressive cells (Tregs, M2 macrophages, and resting dendritic cells) in OV compared to EOC. (b) Stacked bar plot showing the estimated proportions of different immune cell subtypes in OV and EOC samples, highlighting the shift toward proinflammatory immune cells in the OV group. (c) Heat map clustering analysis of immune cell expression patterns, demonstrating that OV samples are enriched in M1 macrophages, CD8+ T cells, and activated mast cells, while EOC samples show a predominance of Tregs and M2 macrophages. (d) Correlation analysis of BST2 expression with immune cell types, showing a positive correlation with Tregs and M2 macrophages and a negative correlation with M1 macrophages and CD8+ T cells, suggesting a regulatory role of BST2 in immune cell infiltration. (e) qRT-PCR analysis of Foxp3 and IL-10 expression in Tregs from SKOV3 and OVCAR3 cells with BST2 knockdown and overexpression. Knockdown of BST2 significantly reduced the expression of Foxp3 and IL-10, while BST2 overexpression increased their levels.

To investigate how BST2 expression shapes the TME, a comparative single-cell analysis was conducted between a general OV cohort and a specific EOC group. This revealed a stark immunological dichotomy: The OV TME was characterized by a proinflammatory infiltrate, rich in M1 macrophages, CD8+ T cells, and activated mast cells. In contrast, the EOC microenvironment was dominated by immunosuppressive populations, including Tregs and M2 macrophages (Figures 6, 6, and 6). Crucially, BST2 expression levels were found to be a key determinant of this immune landscape. Correlation analysis demonstrated that BST2 expression was strongly and positively correlated with the abundance of immunosuppressive Tregs (*r* = 0.53) and M2 macrophages (*r* = 0.32). Conversely, it was negatively correlated with antitumor M1 macrophages (*r* = −0.41) and CD8+ T cells (*r* = −0.45), suggesting that BST2 actively cultivates an immunosuppressive niche (Figure 6). To functionally validate this observation, particularly the link with Tregs, EOC cells with either BST2 knockdown or overexpression were cocultured with isolated Tregs. qRT-PCR analysis of these Tregs revealed that BST2 knockdown in the cancer cells led to a significant downregulation of the key Treg functional markers Foxp3 and IL-10. Conversely, BST2 overexpression in the cancer cells prompted an upregulation of these immunosuppressive factors (Figure 6). Together, these findings demonstrate that BST2 expression is a critical driver of immune evasion in EOC, a mechanism that involves remodeling the TME to favor immunosuppressive cell types and directly potentiating the function of Tregs.

## 4. Discussion

EOC presents a formidable clinical challenge, primarily due to its frequent diagnosis at advanced stages and associated high mortality. Employing the ESTIMATE algorithm and comprehensive bioinformatic analyses, we identified BST2 as a pivotal gene, which we integrated into a predictive risk score. The multifaceted role of BST2 in oncogenesis is well documented across various malignancies, where it modulates processes such as the EMT, the acquisition of drug resistance in CAR T-cell therapies, and the function of tumor-associated macrophages in pancreatic cancer [32–34]. For instance, elevated BST2 expression in glioblastoma multiforme (GBM) correlates with diminished tumor purity, poor prognosis, and altered immune infiltration [35], while in oral squamous cell carcinoma (OSCC), it promotes cellular migration and invasion via the AKT/ERK1/2-STAT1 signaling pathway [36]. Crucially, within the EOC context, the transcription of BST2 is directly regulated by CFP1, linking it to tumor cell proliferation and apoptosis inhibition [22]. The primary contribution of our research is the systematic elucidation of a network where BST2 serves as a central orchestrator within the EOC microenvironment. While the oncogenic roles of neural markers like NES and GFAP in promoting proliferation and invasion have been independently described [37–40], their connection to immune-regulatory molecules in EOC remains enigmatic. Our work demonstrates for the first time that BST2 drives EOC progression through a tripartite mechanism: polarizing macrophages toward a protumoral M2 phenotype, augmenting the immunosuppressive capacity of Tregs, and concurrently upregulating NES and GFAP. This integrated analysis, linking immune modulation with neuromimicry, offers a novel framework for understanding how BST2 engineers a complex ecosystem conducive to tumor growth, invasion, and immune evasion, thereby positioning it as a compelling therapeutic target.

Our investigation commenced with an initial screening of public databases, which identified BST2 as a gene with significant prognostic implications in ovarian cancer. To validate this in silico finding, we assessed BST2 expression in the EOC cell lines SKOV3 and OVCAR3 alongside normal ovarian epithelial cells. Consistent with bioinformatic predictions, BST2 expression was markedly upregulated in the EOC cells, providing an experimental basis for mechanistic inquiry. Subsequent functional assays were designed to directly probe its role. Genetic manipulation to either suppress or overexpress BST2 resulted in significant and corresponding alterations in cellular proliferation, apoptosis, invasion, and migration. These results establish a direct correlation between BST2 expression levels and the malignant phenotype, confirming its function as a key driver of EOC progression.

The active role of the nervous system in modulating oncogenesis is now well established, moving beyond classical histological observations of perineural invasion (PNI) [41]. Preclinical investigations confirm this link, demonstrating that autonomic *β*-adrenergic signaling can accelerate tumor progression in breast and ovarian cancers [42, 43] and that diverse neuronal subtypes are instrumental to proliferation across various malignancies. Furthermore, malignant cells actively orchestrate this neuro-neoplastic interplay. They secrete chemotactic factors that recruit neural progenitors from the brain; these progenitors then migrate through the vasculature and differentiate into new neuronal structures within the tumor, thereby cultivating a protumorigenic niche [44]. Despite these insights, the precise mechanisms governing the integration of peripheral neoplasms into neuronal circuits remain to be fully elucidated.

A key finding from our study is the mechanistic link between BST2 expression and the upregulation of the neural markers NES and GFAP. This axis is highly relevant to EOC progression. NES is a well-recognized marker of cancer stemness associated with increased proliferation, invasiveness, and drug resistance in numerous cancers, including ovarian. Concurrently, GFAP, although a canonical glial marker, facilitates tumor cell migration and invasion by remodeling the cytoskeleton in certain neoplastic contexts. Our data thus unveil a previously unrecognized mechanism: BST2 enhances the malignant EOC phenotype not only via immune modulation but also by fostering a protumorigenic, neuromimetic microenvironment. This upregulation of neural markers provides a direct molecular connection between BST2 and the aggressive cellular behaviors observed in EOC.

The intricate crosstalk between the nervous and immune systems critically shapes the TME. For instance, B lymphocytes have been shown to synthesize the neurotransmitter GABA, which can attenuate the anti-neoplastic activity of CD8+ T cells in colorectal cancer models by engaging GABAA receptors [45]. This complex interplay contributes to the challenges facing modern cancer treatments. While immunotherapies such as CAR T-cell therapy, checkpoint inhibitors, and oncolytic viruses represent formidable approaches [46, 47], their clinical efficacy in EOC has been limited. This therapeutic resistance is largely attributed to the profound cellular and molecular heterogeneity of the EOC TME [48].

Within the TME, TAMs are a dominant cell population, sometimes comprising up to 40% of the immune infiltrate, making them a critical focus of oncological research [49, 50]. TAMs exhibit remarkable plasticity, capable of adopting distinct functional phenotypes in response to microenvironmental signals [51, 52]. They can exert antitumor effects, such as mediating antibody-dependent cytotoxicity and activating adaptive immunity [16, 53]. However, in established tumors, they more frequently acquire a protumorigenic M2-like phenotype that promotes cancer cell proliferation, stimulates angiogenesis, and orchestrates an immunosuppressive niche, thereby facilitating tumor growth and immune evasion [52]. Although BST2 has been identified as a prognostic factor in EOC, its potential to modulate the immune microenvironment and predict immunotherapy response was unknown. Our study addresses this gap. In vivo xenograft experiments revealed that elevated BST2 expression not only accelerated EOC tumor growth but also skewed TAM polarization toward the M2 phenotype. This finding provides a direct mechanism by which BST2 may expedite EOC progression.

Our investigation established that BST2 expression is markedly elevated in EOC tissues and correlates with adverse patient outcomes. A BST2-based prognostic model using TCGA data confirmed that high expression is associated with diminished survival. These clinical findings were substantiated at the cellular level, where BST2 expression was significantly higher in EOC cell lines (SKOV3 and OVCAR3) compared to a normal ovarian epithelial line (IOSE-80). Functionally, BST2 upregulation enhanced proliferation, migration, and invasion, while its suppression reversed these oncogenic traits. These in vitro results were mirrored in vivo, where BST2 overexpression promoted xenograft tumor growth and knockdown had the opposite effect. Crucially, BST2 overexpression also drove M2 macrophage polarization within the EOC tumor tissue. Notably, IHC analysis revealed that BST2 is highly expressed not only in cancer cells but also in nontumor stromal components, aligning with reports of BST2+ macrophages being associated with poor prognosis in pancreatic cancer [31] and BST2 expression in other cell types like stem cells [54].

It is critical to acknowledge the limitations of this study to frame the conclusions appropriately. On the clinical side, our IHC analysis was performed on a small cohort (*n* = 31), which limits statistical power. This is exemplified by the observed trend between higher BST2 expression and advanced FIGO stage, which did not reach statistical significance (*p* = 0.075). Future validation in larger, multicenter cohorts is essential to conclusively establish these clinical associations. Methodologically, the rigor of our findings could be enhanced in subsequent studies. The observed in vivo phenotypes likely represent a composite effect, as BST2 is expressed in both tumor and nontumor cells within the TME; future work using cell-type-specific models is required to dissect these distinct contributions. Similarly, while the use of two independent shRNAs and the opposing gain- and loss-of-function phenotypes provides strong evidence for specificity, the formal inclusion of “rescue” experiments and empty vector controls would definitively confirm these findings and rule out potential off-target effects. Furthermore, while our results suggest the involvement of the IL-4/STAT6 pathway in M2 polarization, further experiments are needed to validate this specific mechanistic link. Our bioinformatic analyses also have inherent limitations; the immune cell proportions were computational estimates derived from the CIBERSORT algorithm, and the comparison of bulk tumor (TCGA) and normal (GTEx) tissues is potentially confounded by baseline differences in cellular composition. Finally, translating these mechanistic findings into clinical applications presents considerable challenges, including the need for tumor-specific targeting strategies, effective drug delivery, and methods to overcome potential therapeutic resistance.

## Figures and Tables

**Table 1:** Correlation between BST2 expression and the clinicopathological features of EOC subtypes: Serous carcinoma and clear cell carcinoma.

**Features**		**Total**	**High BST2 expression, ** **n** ** (%)**	**BST2 low expression, ** **n** ** (%)**	**Pearson's chi-square test ** **p** ** value**
Mean preoperative CA-125	841.65U/mL				
Mean postoperative CA-125	295.99U/mL				
Age (years)	≤53	16	13	3	0.93
	>53	15	12	3	
Histology	Serous carcinoma	21	16 (76.19%)	5 (23.81%)	0.363
	Clear cell carcinoma	10	9 (90%)	1 (10%)	
FIGO	I–II	18	12	6	0.075
	III–IV	13	13	0	
Menopausal status	Yes	21	18	3	0.301
	No	10	7	3	
Weight	≤55	15	13	2	0.411
	>55	16	12	4	
State	CR	25	20	5	0.853
	Recur	6	5	1	

## Data Availability

The data that support the findings of this study are available from the corresponding author upon reasonable request.

## Nomenclature

BST2: Bone Marrow Stromal Antigen 2
EOC: epithelial ovarian cancer
HGSOC: high-grade serous ovarian cancer
qRT-PCR: reverse transcription-quantitative polymerase chain reaction
IHC: immunohistochemistry
TCGA: The Cancer Genome Atlas
M1: classically activated macrophage (proinflammatory)
M2: alternatively activated macrophage (anti-inflammatory)
CCK-8: Cell Counting Kit-8
ELISA: enzyme-linked immunosorbent assay
TME: tumor microenvironment
FBS: fetal bovine serum
PMA: phorbol 12-myristate 13-acetate
GFP: green fluorescent protein
NF-*κ*B: nuclear factor kappa-light-chain-enhancer of activated B cells
ERK1/2: Extracellular Signal–Regulated Kinases 1 and 2
PI: propidium iodide
CA-125: Cancer Antigen 125
FIGO: International Federation of Gynecology and Obstetrics
M0: macrophage phenotype (undifferentiated)
NE: neuron-specific enolase
GFAP: glial fibrillary acidic protein
M1/M2 ratio: ratio of classically activated (M1) to alternatively activated (M2) macrophages
SDS-PAGE: sodium dodecyl sulfate polyacrylamide gel electrophoresis
PVDF: polyvinylidene difluoride
PFA: paraformaldehyde

## Supporting Information

Supporting Information Additional supporting information can be found online in the Supporting Information section. Figure S1: Lentiviral vector information. Figure S1A: LV-0683 pLV-EF1a-BST2-CMV-EGFP-T2A-Puro. Sequencing results: atggcatctacttcgtatgactattgcagagtgcccatggaagacggggataagcgctgtaagcttctgctggggataggaattctggtgctcctgatcatcgtgattctgggggtgcccttgattatcttcaccatcaaggccaacagcgaggcctgccgggacggccttcgggcagtgatggagtgtcgcaatgtcacccatctcctgcaacaagagctgaccgaggcccagaagggctttcaggatgtggaggcccaggccgccacctgcaaccacactgtgatggccctaatggcttccctggatgcagagaaggcccaaggacaaaagaaagtggaggagcttgagggagagatcactacattaaaccataagcttcaggacgcgtctgcagaggtggagcgactgagaagagaaaaccaggtcttaagcgtgagaatcgcggacaagaagtactaccccagctcccaggactccagctccgctgcggcgccccagctgctgattgtgctgctgggcctcagcgctctgctgcagtga. Figure S1B: LV-0684 pLVX-U6-shRNA(BST2)-CMV-Puro. Sequencing results: tgctcctgatcatcgtgattcctcgaggaatcacgatgatcaggagcattttt.
