# Supplementary figures and images for: BST2 Drives Epithelial Ovarian Cancer Progression via Macrophage M2 Polarization, Neural Remodeling, and Immunosuppressive Microenvironment Formation
Source: Hum Mutat. 2025 Nov 13;2025:8719836. doi: 10.1155/humu/8719836 (PMC12634163; doi:10.1155/humu/8719836)

A

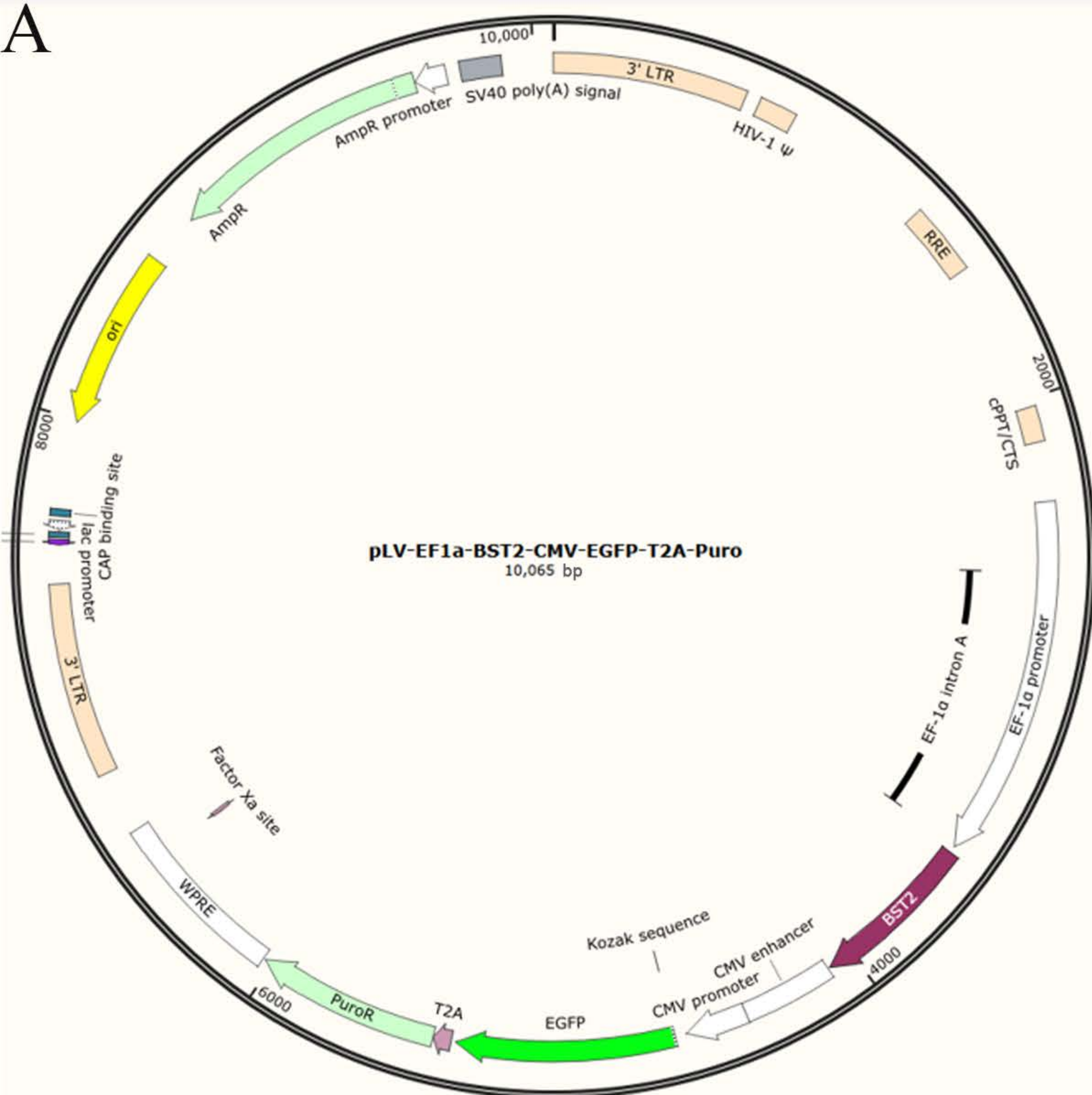

B

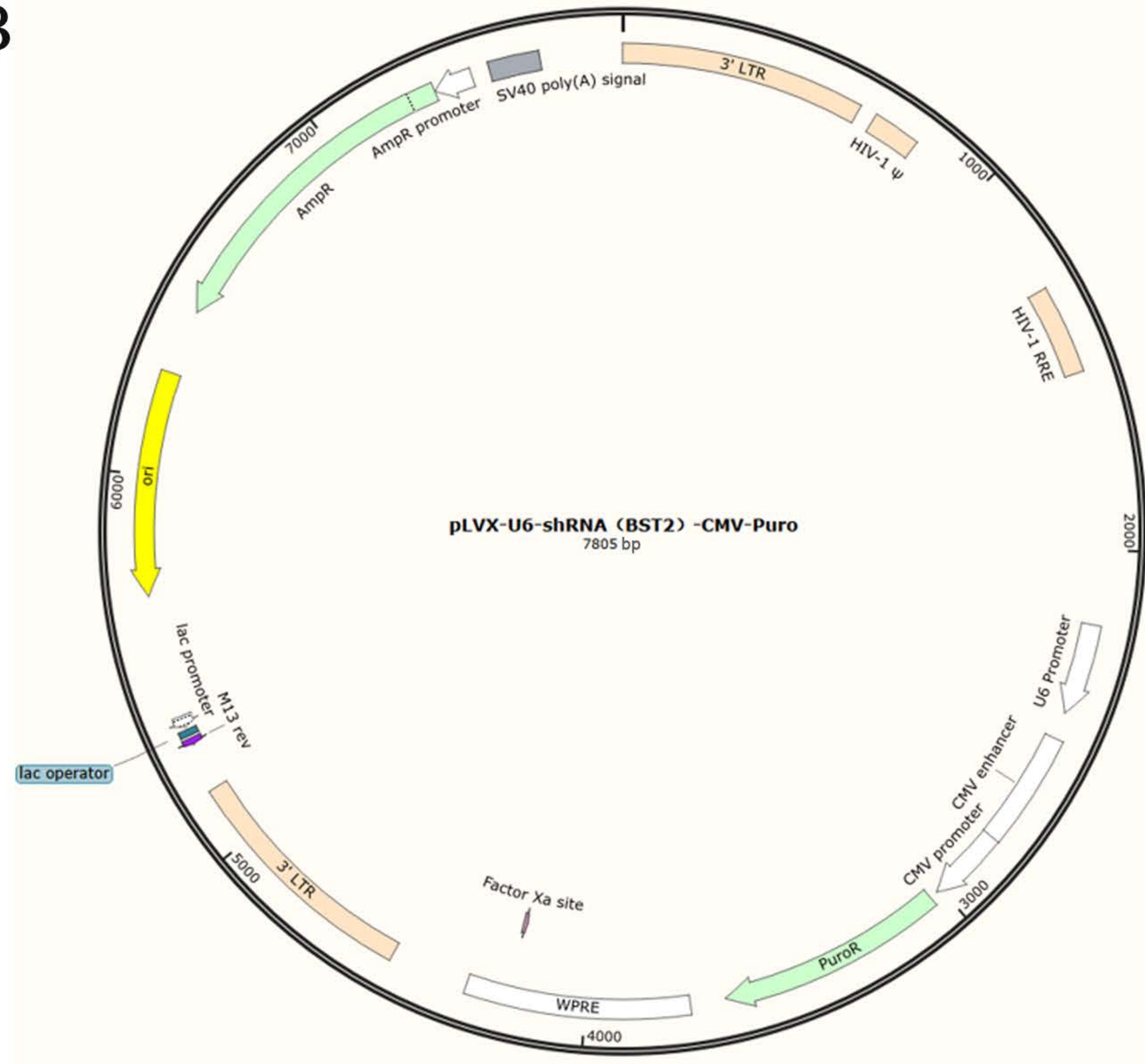

Supplement: Supporting Information — Additional supporting information can be found online in the Supporting Information section. Figure S1: Lentiviral vector information. Figure S1A: LV-0683 pLV-EF1a-BST2-CMV-EGFP-T2A-Puro. Sequencing results: atggcatctacttcgtatgactattgcagagtgcccatggaagacggggataagcgctgtaagcttctgctggggataggaattctggtgctcctgatcatcgtgattctgggggtgcccttgattatcttcaccatcaaggccaacagcgaggcctgccgggacggccttcgggcagtgatggagtgtcgcaatgtcacccatctcctgcaacaagagctgaccgaggcccagaagggctttcaggatgtggaggcccaggccgccacctgcaaccacactgtgatggccctaatggcttccctggatgcagagaaggcccaaggacaaaagaaagtggaggagcttgagggagagatcactacattaaaccataagcttcaggacgcgtctgcagaggtggagcgactgagaagagaaaaccaggtcttaagcgtgagaatcgcggacaagaagtactaccccagctcccaggactccagctccgctgcggcgccccagctgctgattgtgctgctgggcctcagcgctctgctgcagtga. Figure S1B: LV-0684 pLVX-U6-shRNA(BST2)-CMV-Puro. Sequencing results: tgctcctgatcatcgtgattcctcgaggaatcacgatgatcaggagcattttt. [file 8719836.f1.pdf]
